# Acetyl-11-keto-β-boswellic acid (AKBA); targeting oral cavity pathogens

**DOI:** 10.1186/1756-0500-4-406

**Published:** 2011-10-13

**Authors:** Alsaba F Raja, Furqan Ali, Inshad A Khan, Abdul S Shawl, Daljit S Arora

**Affiliations:** 1 Microbiology Unit, Indian Institute of Integrative Medicine (CSIR), Sanatnagar, Srinagar, 190005, India; 2 Clinical Microbiology Division, Indian Institute of Integrative Medicine (CSIR), Canal Road, Jammu, 180001, India; 3 Department of Microbiology, Guru Nanak Dev University, Amritsar Punjab, 143005, India

**Keywords:** *Streptococcus mutans*, Biofilm, PAE, *Boswellia serrata*

## Abstract

**Background:**

Boswellic acids mixture of triterpenic acids obtained from the oleo gum resin of *Boswellia serrata *and known for its effectiveness in the treatment of chronic inflammatory disease including peritumor edema. Boswellic acids have been extensively studied for a number of activities including anti inflammatory, antitumor, immunomodulatory, and inflammatory bowel diseases. The present study describes the antimicrobial activities of boswellic acid molecules against oral cavity pathogens. Acetyl-11-keto-β-boswellic acid (AKBA), which exhibited the most potent antibacterial activity, was further evaluated in time kill studies, mutation prevention frequency, postantibiotic effect (PAE) and biofilm susceptibility assay against oral cavity pathogens.

**Findings:**

AKBA exhibited an inhibitory effect on all the oral cavity pathogens tested (MIC of 2-4 μg/ml). It exhibited concentration dependent killing of S*treptococcus mutans *ATCC 25175 up to 8 × MIC and also prevented the emergence of mutants of *S.mutans *ATCC 25175 at 8× MIC. AKBA demonstrated postantibiotic effect (PAE) of 5.7 ± 0.1 h at 2 × MIC. Furthermore, AKBA inhibited the formation of biofilms generated by *S.mutans *and *Actinomyces viscosus *and also reduced the preformed biofilms by these bacteria.

**Conclusions:**

AKBA can be useful compound for the development of antibacterial agent against oral pathogens and it has great potential for use in mouthwash for preventing and treating oral infections.

## Background

Several microorganisms inhabit the human oral cavity, and there is always a risk of infection with bacterial pathogens associated with the oral cavity. *Streptococcus *constitutes 60 to 90% of the remaining bacteria that colonize the teeth within the first 4 h after professional cleaning [1]. Other early colonizers include *Actinomyces *spp., *Eikenella *spp., *Haemophilus *spp., *Prevotella *spp., *Propionibacterium *spp., and *Veillonella *spp. Many of the physical interactions that occur between the organisms of this community are known. *Streptococcus *is the only genus of oral cavity bacteria that demonstrates extensive and intergenic coaggregation [2,3]. The ability of this genus to bind to other early colonizers and to host oral matrices may confer an opportunity to viridians streptococci in establishing early dental plaque [1]. *Streptococcus mutans *can colonize the tooth surface and initiate plaque formation by its ability to synthesize extracellular polysaccharides, mainly water-insoluble glucan from sucrose, using its glucosyltransferase [4]. It is a key contributor to the formation of biofilms associated with dental caries disease. The biofilms of *S. mutans *are also involved in infective endocarditis, a serious disease with a mortality rate of up to 50% despite antibiotic treatment [5]. The current research targeting microbial biofilm inhibition has attracted a great deal of attention, and the search for effective antimicrobial agents against these oral pathogens could lead to identification of new agents for the prevention of dental caries and periodontal diseases arising out of dental plaque formation [6]. A variety of plant materials and phytochemicals, especially a class of essential oils, have long been found to exhibit effective antibacterial activity [7]. The aromatic molecules derived from natural sources are being explored extensively as alternative agents in oral care products. There is some evidence that many natural molecules are good antibacterial agents that show activity against oral pathogens like *Fusobacterium nucleatum*, *Actinomyces viscosus*, *S. mutans*, *Prevotella intermedia*, *Haemophilus actinomycetemcomitans*, *Streptococcus sanguis *and *Prophyromonas gingivalis *[8-11].

Boswellic acids, major constituents of the gum resin derived from the plant *Boswellia serrata*, comprises of β-boswellic acids as the main triterpenic acid along with 11-keto-β-boswellic acids and their acetates [12]. The gum exudate is known for its anti-inflammatory properties in the Ayurvedic system of medicines [13,14]. The alcoholic extract of the gum is used for the treatment of adjuvant arthritis [15]. It has synergistic effect with glucosamine, an anti-inflammatory and anti-arthritic agent [16]. Acetyl-11-keto-β-boswellic acid (AKBA), a component of the gum exudate is a pentacyclic terpenoid and is reported to be active against a large number of inflammatory diseases [17,18] including cancer, arthritis, chronic colitis, ulcerative colitis, Crohn's disease, and bronchial asthma [19-21]. In addition to these therapeutic effects, our recent studies have revealed antibacterial properties of AKBA against various clinical isolates and ATCC strains of Gram positive bacteria [22]. The aim of study was to evaluate the antibacterial activity of acetyl-11-keto-β-boswellic acid against a panel of oral cavity pathogens and its biofilm inhibitory potential for *Streptococcus mutans *ATCC 25175 (cariogenic bacteria) and *Actinomyces viscocsus *ATCC 15987 (noncariogenic bacteria).

## Methods

### Extraction and isolation of boswellic acid molecules from gum resin of *Boswellia serrata*

β-boswellic acid (BA), 11-keto-β-boswellic acid (KBA), Acetyl-β-boswellic acid (ABA) and acetyl-11-keto-β-boswellic acid (AKBA) were obtained from Bioorganic Chemistry Division of Indian Institute of Integrative Medicine Jammu, India. The extraction, isolation, and quantification of these compounds from gum resin of *Boswellia serrata *were described in our previous study [16,23].

### Bacterial strains and culture conditions

The pathogenic bacterial strains were obtained from ATCC (American Type Culture Collection, Manassas, VA, USA). *Streptococcus mutans *ATCC 25175, *Enterococcus faecalis *ATCC 29212, *Enterococcus faecium *ATCC 8042 were maintained by sub culturing on Tryticase Soy agar (TSA; DIFCO Laboratories, Detroit, MI, USA) at 37°C. Cultures of *Actinomyces viscosus *ATCC 15987 and *Streptococcus sanguis *ATCC 10556 were maintained on Brain heart infusion agar (BHI; DIFCO Laboratories) at 37°C in a 5% CO_2 _atmosphere. *Porphyromonas gingivalis *ATCC 33277, *Fusobacterium nucleatum *ATCC 10953, and *Prevotella intermedia *ATCC 25611 were maintained on Wilkins Chalgren agar (WCA; DIFCO Laboratories) in an anaerobic gas jar at 37°C.

### Minimum inhibitory concentrations (MIC) and minimum bactericidal concentrations (MBC) of boswellic acids against oral cavity pathogens

MIC was determined as per the guidelines of Clinical and Laboratory Standards Institute (formerly the National Committee for Clinical Laboratory Standards) [24]. All oral cavity bacteria used in this study were grown to stationary phase for 24 h at 37°C. Bacterial suspensions were prepared by suspending 24 h grown culture in Brucella broth (BB; DIFCO Laboratories) (for anaerobic bacteria) and sterile normal saline (0.89% NaCl wt/vol; Himedia, Mumbai India, for aerobic bacteria). The turbidity of bacterial suspension was adjusted to 0.5 McFarland standard, which is equivalent to 1.5 × 10^8 ^CFU/ml. The boswellic acids stock solutions were prepared in 100% dimethyl sulfoxide (DMSO; Merck, Mumbai India) and 2-fold serial dilutions were prepared in Mueller Hinton Broth (MHB; Difco Laboratories) for aerobic bacteria, Brain heart infusion broth (BHI) for 5% CO_2 _cultures and WCB for anaerobic bacteria) respectively in 100 μl volume in 96-well U bottom microtiter plates (Tarson, Mumbai, India). The above-mentioned bacterial suspension was further diluted in respective growth media and 100 μl volume of this diluted inoculum was added to each well of the plate resulting in the final inoculum of 5 × 10^5 ^CFU/ml in the well and the final concentration of boswellic acids ranged from 0.25 to 128 μg/ml. Triclosan was used as standard antibacterial agent for this study at a concentration ranged from 0.03-16 μg/ml. The plates were incubated at 37°C for 24 h and were visually read for the absence or presence of turbidity. The minimum concentration of the compound concentration showing no turbidity was recorded as MIC. The MBC was determined by spreading 100 μl volume on tryptic soy agar (TSA) plate from the wells showing no visible growth. The plates were incubated at 37°C for overnight.

### Time kill studies against *S. mutans*

*S. mutans *ATCC 25175 was grown in BHI broth at 37°C for 24 h. The turbidity of the suspension was adjusted to 0.5 McFarland (≈ 1.5 × 10^8 ^CFU/ml) in sterile normal saline. Two hundred microliters of this suspension was used to inoculate 20 ml of BHI broth conical flask containing AKBA in the concentration range of 8-32 μg/ml. DMSO controls were also included in the study. The flasks were incubated at 37°C. One hundred microliters samples were taken at 0, 1, 2, 4, 6, 8, 10, and 24 h and the viable counts were determined in triplicate on TSA. Killing curves were constructed by plotting the log_10 _CFU/ml versus time over 24 h [25].

### Selection of resistant mutants *in vitro*

The first-step mutants of *S. mutans *ATCC 25175 were selected using a previously described method [26]. A bacterial suspension containing 10^9 ^CFU (100 μl) was plated on BHI agar containing AKBA at concentrations equal to 2×, 4×, and 8× MIC. Mutation frequency was calculated by counting the total number of colonies appearing after 48 h of incubation at 37°C in 5% CO_2 _on the AKBA-containing plate and by dividing the number by the total number of CFU plated. All mutation prevention concentration determinations were made in triplicate, and the results were identical.

### Postantibiotic effect (PAE)

The PAEs of the AKBA were assessed by the method described by Craig and Gudmundsson [27]. AKBA was added at the MIC and 2 × MIC to test tubes containing ≈10^6 ^CFU/ml of *S. mutans *ATCC 25175 in BHI broth. After an exposure of 2 h to the AKBA, samples were diluted to 1:1,000 in same medium to effectively remove AKBA. CFU was determined from the sample every hour until visual cloudiness was noted. The PAE was calculated by the equation: PAE = *T - C*, where *T *represents the time required for the count in the test culture to increase 1 log_10 _CFU/ml above the count observed immediately after drug removal and *C *represents the time required for the count of the untreated control tube to increase by 1 log_10 _CFU/ml.

### Biofilm susceptibility assays

The biofilms of *S. mutans *ATCC 25175 and *A. viscosus *ATCC 15987 were prepared in 96-well flat-bottom polystyrene microtiter plates (Tarson, Mumbai, India), using a previously described method of Wei et al. [28] with a few modifications. This method was similar to the MIC assay for planktonic cells. The bacterial suspensions were prepared from the overnight grown culture and the turbidity of the suspension was adjusted to 0.7 O.D.610 (≈1 × 10^9 ^CFU/ml). Twofold serial dilutions of boswellic acids were prepared in 100 μl volume in BHI supplemented with 2% sucrose in the wells of 96-well flat bottom microtiter plate. Forty microliters of fresh BHI with 2% sucrose was added to each well, followed by the addition of 60 μl of above bacterial suspension. This resulted in the final inoculum of 6 × 10^7 ^CFU/ml in each well: the final concentrations of the compounds ranged from (0.12 to 128 μg/ml). The plate was incubated for 18 h at 37°C in 5% CO2 for 24 h, absorbance at 595 nm was recorded to assess the culture growth. After completion of incubation, the planktonic cells were removed from each well by washing with phosphate buffer saline (Himedia, Mumbai, India). The biofilms were fixed with methanol for 15-30 min, stained with 0.1% (wt/vol) Crystal Violet (Sigma Chemical Co., St Louis, MO, USA). The biofilm was rinsed thoroughly with water until the control wells appeared colourless. Biofilm formation was quantified by the estimation of biofilm mass (glucans matrix containing bacterial cells) with the addition of 200 μl of 95% ethanol to each Crystal Violet-stained well. The plate was put on a shaker at room temperature for 30 min and the absorbance at 595 nm (A_595_) was determined using a microplate reader (Multiskan Spectrum; Thermo Electron, Vantaa, Finland). The percentage of inhibition was calculated using the equation (1-A_595 _of the test/A_595 _of nontreated control) ×100. Culture without the agents was used as the no-treatment control. The minimum biofilm inhibition concentration (MBIC_50_) was defined as the lowest agent concentration that showed 50% or more inhibition on the formation of biofilm.

The effect of AKBA was also examined on preformed biofilm. The biofilms were prepared by inoculating the suspension of *S. mutans *ATCC 25175 and *A. viscosus *ATCC 15987 into the wells of a polystyrene microtiter plate as mentioned above. After incubation at 37°C in 5% CO2 for 24 h, the culture supernatant from each well was decanted, and the planktonic cells were removed by washing the wells with PBS (pH 7.2). Twofold serial dilutions of AKBA were prepared in BHI broth, and 200 μl of each dilution was added to the biofilm in the wells. The plate was further incubated at 37°C in 5% CO_2 _for 24 h. The biofilm was fixed, stained, and quantified as described above.

### Statistical analysis

All experiments were carried out in triplicates in at least three different occasions. Differences between two means were evaluated by the Student's *t*-test. The data were analyzed by one-way ANOVA for comparison of multiple means followed by Bonferroni test using GraphPad Instat2 program (GraphPad software Inc. San Diego CA). The chosen level of significance for all statistical tests was *P *< 0.05.

## Results

### MIC and MBC of boswellic acids

The *in vitro *antibacterial activities of boswellic acids were tested on a group of clinically significant panel of oral bacteria (Table 1). AKBA was the most active of the four boswellic acids against all bacterial pathogens. AKBA exhibited MIC ranging from 2-4 μg/ml against all the tested strains except against *F. nucleatum *ATCC 10953 showing MIC > 128 μg/ml, whereas KBA and BA exhibited moderate Gram-positive antibacterial activity (MIC ≈ 8-64 μg/ml). ABA on the other hand was completely devoid of antibacterial activity up to the tested concentration of 128 μg/ml. All the compounds were bacteriostatic in nature and exhibited an MBC > 128 μg/ml. Since AKBA was found to be the most active boswellic acid compound against Gram-positive bacterial pathogens, further *in vitro *studies were performed on this compound against clinically important *S. mutans *and *A. viscosus*.

**Table 1:** Antibacterial activity of boswellic acid molecules against Oral pathogens.

Organisms		KBA	AKBA	BA	ABA	
		
	Triclosan	MIC^a^	MIC^a^	MIC^a^	MIC^a^	MBC^b^
*S. mutans *ATCC 25175	4	16	2	32	> 128	> 128
*E. faecalis *ATCC 29212	4	16	4	8	> 128	> 128
*E. faecium *ATCC 8042	4	16	4	8	> 128	> 128
*A. viscosus *ATCC 15987	4	8	2	64	> 128	> 128
*S. sanguinis *ATCC 10556	4	8	2	128	> 128	> 128
*F. nucleatum *ATCC 10953	2	> 128	> 128	> 128	> 128	> 128
*P. intermedia *ATCC 25611	1	16	4	32	> 128	> 128
*P. gingivalis *ATCC 33277	2	8	4	32	> 128	> 128

MICs and MBCs of boswellic acid molecules were determined using CLSI guidelines against ATCC strains. ^a^Minimum Inhibitory Concentration (μg/ml); ^b^Minimum Bactericidal Concentration (μg/ml);

### Time-kill kinetic studies

**Figure 1:**
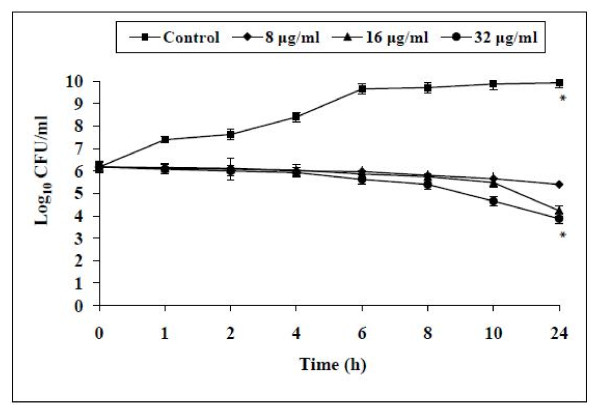
**Effect of AKBA at different concentrations (8, 16 and 32 μg/ml) on the cell viabilty of *S.mutans *ATCC 25175**. *S. mutans *cells without AKBA served as control. The effect of AKBA was observed bacteriostatic at all tested concentrations when compared with non treated control (*P *< 0.05) over a period of 24 h study. Each time point represents the mean log_10 _standard deviations (± SD) of three different experiments performed in duplicate. *, *P <*0.05; (Student's *t *test).

The time-kill kinetics studies were specifically performed against *S. mutans *ATCC 25175 owing to its importance in the initiation of plaque formation (Figure 1). It showed bacteriostatic activity at all the tested concentrations. The maximum effect of AKBA was observed at 16 and 32 μg/ml exhibiting a ≈2 log_10 _reduction in the viability of *S. mutans *cells when compared with non treated controls (*P *< 0.05) at four and eight times it's MIC over a period of 24 h study.

### Frequency of emergence of AKBA resistance

The frequencies of mutant selection of *S. mutans *ATCC 25175 are shown in table 2. AKBA at 16 μg/ml (8 × MIC) completely suppressed the emergence of mutants. This concentration of AKBA at which no mutant was selected can be defined as the mutation prevention concentration.

**Table 2:** Frequency of mutation with Acetyl-11-keto-β-boswellic acid against *S.mutans *ATCC 25175.

Compounds	Mutation frequency with AKBA at:^a^		
	
	2 × MIC	4 × MIC	8 × MIC
Acetyl-11-keto-β-boswellic acid	3 × 10^-9^	3 × 10^-9^	< 3 ×10^-9^
Ciprofloxacin	2.5 × 10^-6^	3.5 × 10^-8^	1.5 × 10^-9^

^a ^The MIC of Acetyl-11-keto-β-boswellic acid is 2 μg/ml.

MPC were performed on *S. mutans *ATCC 25175. The rate of mutation frequency was calculated by dividing total number of colonies appearing on the drug containing plate by total number of CFU plated. AKBA at 16 μg/ml (8 × MIC) completely suppressed the emergence of mutants. This concentration of AKBA at which no mutant was selected can be defined as the mutation prevention concentration (MPC).

### Postantibiotic Effect (PAEs)

The PAE of AKBA was determined on *S. mutans *(Table 3). The PAE induced by AKBA was concentration dependent, with duration 3.5 ± 0.1 h at 1 × MIC while at 2 × MIC it was 5.7 ± 0.1 h. Ciprofloxacin was used as control drug in the study and it exhibited a PAE of 1.4 ± 0.05 h at 1 × MIC while at 2 × MIC it was 2.2 ± 0.1 h (0.5 μg/ml). The PAEs of AKBA were significantly higher than the ciprofloxacin against *S. mutans *(*P *< 0.05).

**Table 3:** PAEs of Acetyl-11-keto-β-boswellic acid against *S.mutans *ATCC 25175.

Compounds	Mean PAE (h) ± SD on:	
	
	1 × MIC	2 × MIC
Acetyl-11-keto-β-boswellic acid	3.5 ± 0.1^a^	5.7 ± 0.1^b^
Ciprofloxacin	1.4 ± 0.05^a^	2.2 ± 0.1^b^

The PAEs were monitored by viable count of *S. mutans *after 2 h exposure to concentrations equal to MIC and 2 × MIC of antimicrobials (AKBA and ciprofloxacin). Values in the same column followed by the same superscripts are significantly different from each other (*P *< 0.05; Student's *t *test). PAE, Post antibiotic effect.

### Biofilm inhibition and reduction

**Figure 2:**
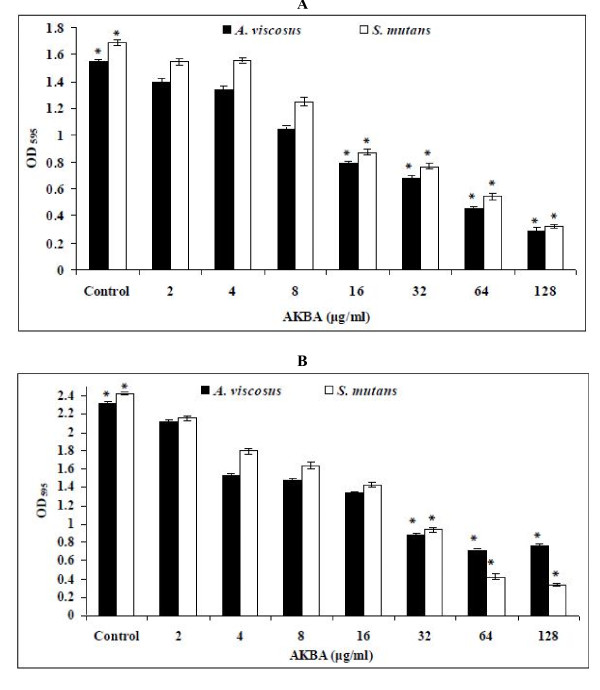
**Effect of AKBA on the biofilm formation (A) and preformed biofilm (B) by *S. mutans *ATCC 25175 and *A. viscosus *ATCC 15987**. After incubation, the biofilms were stained with crystal violet and the optical density of stained biofilm was determined with a multidetection microplate reader at a wavelength of 595 nm (OD_595_). The results are expressed as average optical density readings for crystal violet assays compared to untreated control (without AKBA). The biofilm of *S. mutans *and *A. viscosus *were significantly inhibited (A) and reduced (B) when compared with untreated control (*P *< 0.05). Values are mean (± SD) from four independent determinations. *, *P <*0.05 (Student's *t *test).

AKBA effectively inhibited the formation of *S. mutans *and *A. viscosus *biofilms, with 50% biofilm inhibition concentration (MBIC_50_) 16 μg/ml (as derived from Figure 2A) which is in the range of 8 × MIC. AKBA also effectively eradicated the preformed biofilms. The 50% biofilm reduction concentration (MBRC_50_) ranged 32 μg/ml for both the bacterial isolates (Figure 2B).

## Discussion and conclusions

Boswellic acid obtained from the bark of *Boswellia serrata *was studied for its inhibitory activity against oral cavity pathogens. The *in vitro *antibacterial activity results of four boswellic acid compounds revealed AKBA to be the most potent antibacterial compound against all the bacteria tested including *S. mutans*, *E. faecium*, *E. faecalis*, *S. sanguis*, *A. viscosus*, *P. intermedia *and *P. gingivalis*. AKBA exerted bacteriostatic antibacterial activity against *S. mutans *(Figure 1) and exhibited a good PAE of 5.7 h at 2 × MIC concentration. AKBA at 8 × MIC also prevented the emergence of mutants of *S. mutans *and *A. viscosus*.

Bacteria in a biofilm are invariably less susceptible to antimicrobial agents than their planktonic counterparts [29]. Biofilm infections are difficult to treat due to their inherent antibiotic resistance [30-32]. Oral biofilms are associated with the most common infections in the oral cavity such as caries, gingivitis and periodontal diseases [31]. Oral microbial-plaque communities are biofilms composed of numerous genetically distinct types of bacteria that live in close juxtaposition on host surfaces. These bacteria communicate through physical interactions called coaggregation and coadhesion, as well as other physiological and metabolic interactions [2,3]. The early colonizers namely *Streptococcus mutans *and *Actinomyces viscosus *(mainly from Gram-positive bacteria) initiate the process of acid formation, its deposition and subsequent action on the enamel of the teeth which sets in the process of decalcification and development of dental caries [1,33,34]. AKBA effectively inhibited the *S. mutans *(cariogenic bacteria) and *A. viscosus *(noncariogenic bacteria) biofilms and also reduced the preformed biofilm of these bacterial pathogens (*P *< 0.05). To our knowledge, this is the first report to provide the evidence that AKBA can prevent as well as reduce the *S. mutans *and *A. viscosus *generated biofilms.

In our previous study, we have reported the first time AKBA as the single most potent antibacterial compound present in the gum exudates of *Boswellia serrata *[22], and in this study, first we are reporting AKBA as an antibacterial and antibiofilm agent against oral cavity pathogens. AKBA is reported to be active against a large number of inflammatory diseases, cancer, arthritis, chronic colitis, ulcerative colitis, Crohn's disease, and bronchial asthma [19,20,35-37]. The anticancer activity of AKBA is attributed to the inhibitory effect on the lipoxygenases leading to the inhibition of cell proliferation and induction of apoptosis in tumor cells [38]. There are numerous reports available on the antibacterial activity of oleo-gum resin extracts and oleo-gum resin essential oils from *Boswellia *spp. (Burseraceae) [39-41]. Weckessera et al. [42] reported the antibacterial activity of Boswellia dry extract and keto-β-boswellic acid. Their findings revealed that the extract was highly effective against selected aerobic and anaerobic bacteria such as *Streptococcus, Corynebacteria, C. perfringens *and *P. acnes*; whereas KBA was not effective against these pathogens, suggesting that the effective components are other boswellic acids or essential oils contained in the extract.

Gum resin of *boswellia *is included in the list of substances Generally Recognized As Safe (GRAS), thereby permitting its use as food additive by US FDA. Boswellic acid extract and AKBA have also been reported to be safe and exert minimal toxicity on human skin cells [43]. The recent study indicates that *B. serrata *is non-mutagenic in Ames test, and is non-clastogenic in *in-vitro *chromosomal aberration study [44]. Oral preparations of *Boswellic serrata *extract containing AKBA are sold in the market as over the counter (OTC) anti-inflammatory formulations and are considered to be quite safe [45]. The ancient Indian system of medicine (Ayurveda) claims these preparations to be safe and effective dietary supplement against joint disorders [46,13,14]. Preliminary pharmacokinetic studies carried out in humans yielded low concentrations of boswellic acids in plasma [47-49]. In addition to the above reported usage and safety associated with AKBA, the potent antibacterial and anti-biofilm activities reported in this study warrants that the structure of AKBA can be further exploited to evolve potential lead compounds in the discovery of oral care agents.

## Competing interests

The authors declare that they have no competing interests.

## Authors' contributions

AFR: principle investigator, conceived of the studies, designed the studies and, performed statistical analyses and manuscript writing. FA & IAK contributed substantively to the work and manuscript writing. DSA & ASS: provided valuable comments to the paper in general and was involved in drafting the manuscript. All authors read and approved the final manuscript.
